# Comparative Analysis of CREB3 and CREB3L2 Protein Expression in HEK293 Cells

**DOI:** 10.3390/ijms22052767

**Published:** 2021-03-09

**Authors:** Kentaro Oh-hashi, Ayumi Yamamoto, Ryoichi Murase, Yoko Hirata

**Affiliations:** 1 United Graduate School of Drug Discovery and Medical Information Sciences, Gifu University, 1-1 Yanagido, Gifu 501-1193, Japan; yokoh@gifu-u.ac.jp; 2 Graduate School of Natural Science and Technology, Gifu University, 1-1 Yanagido, Gifu 501-1193, Japan; 3 Department of Chemistry and Biomolecular Science, Faculty of Engineering, Gifu University, 1-1 Yanagido, Gifu 501-1193, Japan; w3032146@edu.gifu-u.ac.jp (A.Y.); w3032137@edu.gifu-u.ac.jp (R.M.)

**Keywords:** CREB3, CREB3L2, ERAD, SEL1L

## Abstract

We performed a comparative analysis of two ER-resident CREB3 family proteins, CREB3 and CREB3L2, in HEK293 cells using pharmacological and genome editing approaches and identified several differences between the two. Treatment with brefeldin A (BFA) and monensin induced the cleavage of full-length CREB3 and CREB3L2; however, the level of the full-length CREB3 protein, but not CREB3L2 protein, was not noticeably reduced by the monensin treatment. On the other hand, treatment with tunicamycin (Tm) shifted the molecular weight of the full-length CREB3L2 protein downward but abolished CREB3 protein expression. Thapsigargin (Tg) significantly increased the expression of only full-length CREB3L2 protein concomitant with a slight increase in the level of its cleaved form. Treatment with cycloheximide and MG132 revealed that both endogenous CREB3 and CREB3L2 are proteasome substrates. In addition, kifunensine, an α-mannosidase inhibitor, significantly increased the levels of both full-length forms. Consistent with these findings, cells lacking SEL1L, a crucial ER-associated protein degradation (ERAD) component, showed increased expression of both full-length CREB3 and CREB3L2; however, cycloheximide treatment downregulated full-length CREB3L2 protein expression more rapidly in SEL1L-deficient cells than the full-length CREB3 protein. Finally, we investigated the induction of the expression of several CREB3 and CREB3L2 target genes by Tg and BFA treatments and SEL1L deficiency. In conclusion, this study suggests that both endogenous full-length CREB3 and CREB3L2 are substrates for ER-associated protein degradation but are partially regulated by distinct mechanisms, each of which contributes to unique cellular responses that are distinct from canonical ER signals.

## 1. Introduction

The ATF6/CREB3 family possesses a similar structure: an N-terminal transactivation region, a basic leucine zipper structure (bZIP), and a single-pass transmembrane region [1,2,3,4,5,6,7,8]. Among these proteins, ATF6 is a canonical ER resident stress sensor, and the transcriptional regulation of a variety of ATF6-regulating genes has been well characterized [3,9]. Regarding the CREB3 family, CREB3 and four other CREB3-like proteins (CREB3L1-4) have been identified to date [4,5,6,7,8]. Immunocytochemical analyses of endogenous and transfected CREB3 family members showed that each full-length form predominantly localizes within the ER [6,7,8,10]. Therefore, CREB3 family members and ATF6 are presumed to be processed by the specific regulated intramembrane proteolysis (RIP) pathway [1,2,3,4,5,6,7,8,11]. CREB3, also called Luman and LZIP, was first identified as a factor associated with herpes simplex virus-related host cell factor 1 (HCF1) [4,12]. The CREB3/HCF1 complex localizes to the nucleus and regulates viral reactivation. CREB3L2 is also called BBF2H7 and is suggested to regulate the secretory capacity of the ER [6,13,14,15,16]. To date, some CREB3 and CREB3L2 target genes have been reported; however, most genes regulated by CREB3 and CREB3L2 remain to be determined.

Recently, we reported the expression and subcellular localization of endogenous and transfected CREB3 using pharmacological and genome editing approaches [10,17]. Based on our results, we simultaneously investigated endogenous CREB3 and CREB3L2 protein expression in HEK293 cells and found that both are unstable and SEL1L-regulated ER-associated protein degradation (ERAD) substrates, although their stabilities are not comparable [18,19,20]. Interestingly, reagents triggering the cleavage of CREB3 and CREB3L2 differed from the well-used ER stress-inducing reagents. In particular, the effects of thapsigargin (Tg), tunicamycin (Tm), and monensin on the expression of each full-length protein were not the same. Finally, we investigated the expression of several CREB3 and CREB3L2 target genes in wild-type and SEL1L-deficient cells. Taken together, studies with SEL1L-deficient cells and reagents affecting ER homeostasis showed that both full-length CREB3 and CREB3L2 are ERAD substrates that are regulated by distinct mechanisms.

## 2. Results and Discussion

Both CREB3 and CREB3L2 are type II ER transmembrane bZIP transcription factors, and their structures are similar to ATF6 [1,2,3,4,6]. Studies analyzing the overexpression of each ATF6/CREB3 family member revealed that they are predominantly localized within the ER and that their cleaved N-terminal halves are transported into the nucleus [6,7,8,10,12]. Among these proteins, ATF6 is the best characterized and an ER stress-responsive transducer, and various ATF6-regulated genes have been identified [3,9]. In addition, the biosynthesis and processing of endogenous ATF6 have been reported in several cell types [21,22,23]. Recently, we documented the expression and subcellular localization of endogenous and transfected CREB3 using pharmacological and genome editing approaches and found that the stimuli triggering CREB3 cleavage are distinct from canonical ER stress-inducing stimuli [10,17]. Our studies also showed that CREB3 expression was largely regulated in a posttranscriptional manner. Based on this knowledge, we tried to compare the expression and processing of endogenous CREB3 and CREB3L2 proteins using several types of reagents and SEL1L1-deficient HEK293 cells.

**Figure 1:**
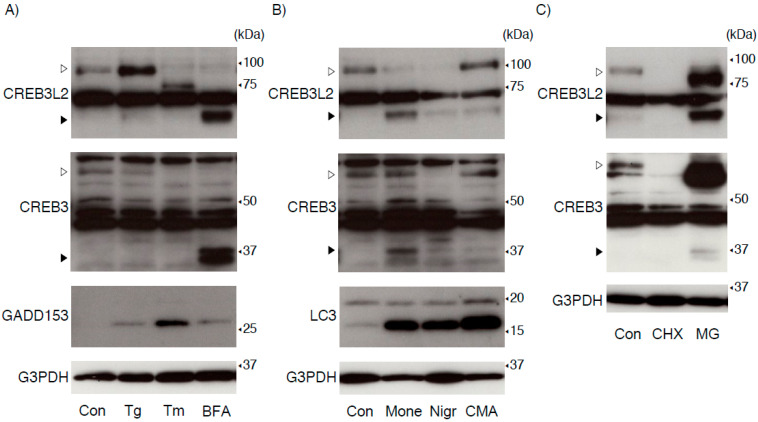
Expression of the CREB3 and CREB3L2 proteins in HEK293 cells. (**A**–**C**) HEK293 cells were treated with thapsigargin (Tg, 0.01 μM), tunicamycin (Tm, 2 μg/mL), brefeldin A (BFA, 0.5 μg/mL), monensin (Mone, 1 μM), nigericin (Nigr, 0.2 μM), concanamycin A (CMA, 50 nM), cycloheximide (CHX, 20 μg/mL), MG132 (MG, 10 μM), or vehicle (Control (Con)) for 8 h. The expression of the indicated protein was detected as described in the Materials and Methods. Open and filled arrowheads indicate full-length mature and cleaved CREB3 and CREB3L2 proteins, respectively. Representative results of three independent cultures are shown.

First, we evaluated the expression of endogenous CREB3 and CREB3L2 proteins in HEK293 cells after treatment with three well-known ER stress inducers: thapsigargin (Tg), tunicamycin (Tm), and brefeldin A (BFA) (Figure 1A). These treatments induced the expression of GADD153 protein, a typical ER stress-inducible factor [24]. Both full-length CREB3 and CREB3L2 disappeared following BFA treatment, in parallel to the remarkable increase in the level of each cleaved form. These cleaved forms are derived from the N-terminal regions of full-length CREB3 and CREB3L2, which protrude from the ER membrane and are transported into the nucleus to induce target gene transcription [1,2]. On the other hand, the effects of the other two inducers on their expression were quite different. Tg treatment apparently increased the level of the CREB3L2 protein, but not that of the CREB3 protein, accompanied by a slight increase in the cleaved CREB3L2 level, although Tg-induced CREB3L2 cleavage occurred at a much lower level than BFA-induced CREB3L2 cleavage. Tm treatment shifted the molecular weight of full-length CREB3L2 downward, due to the inhibition of N-glycosylation by Tm. In contrast, full-length CREB3 disappeared after Tm treatment, and Tm did not increase the level of each cleaved form. Treatment with monensin, nigericin, or concanamycin A dramatically resulted in LC3-II accumulation due to inhibition of the lysosomal pathway; however, the apparent increase in the levels of cleaved CREB3 and CREB3L2 was observed only in cells treated with monensin (Figure 1B). Interestingly, the monensin treatment remarkably downregulated full-length CREB3L2 in HEK293 cells. On the other hand, the nigericin treatment almost completely depleted both full-length CREB3 and CREB3L2. Recently, a novel stress pathway disrupting Golgi structures and functions has been proposed and called Golgi stress [25,26,27], although precise molecular mechanisms remain unclear compared with ER stress [3,9,28,29]. BFA, monensin, and nigericin are often used to trigger Golgi stress through their distinct pharmacological actions, and the effects of these reagents on the expression and processing of CREB3 and CREB3L2 are quite different. These results therefore imply that the CREB3 and CREB3L2 proteins are not regulated by the same molecular mechanisms. On the other hand, the negligible effects of concanamycin A, a V-ATPase inhibitor [30,31], on each protein indicate that autophagy/lysosomal disorders are not directly associated with the Golgi stress pathway, including CREB3 and CREB3L2.

**Figure 2:**
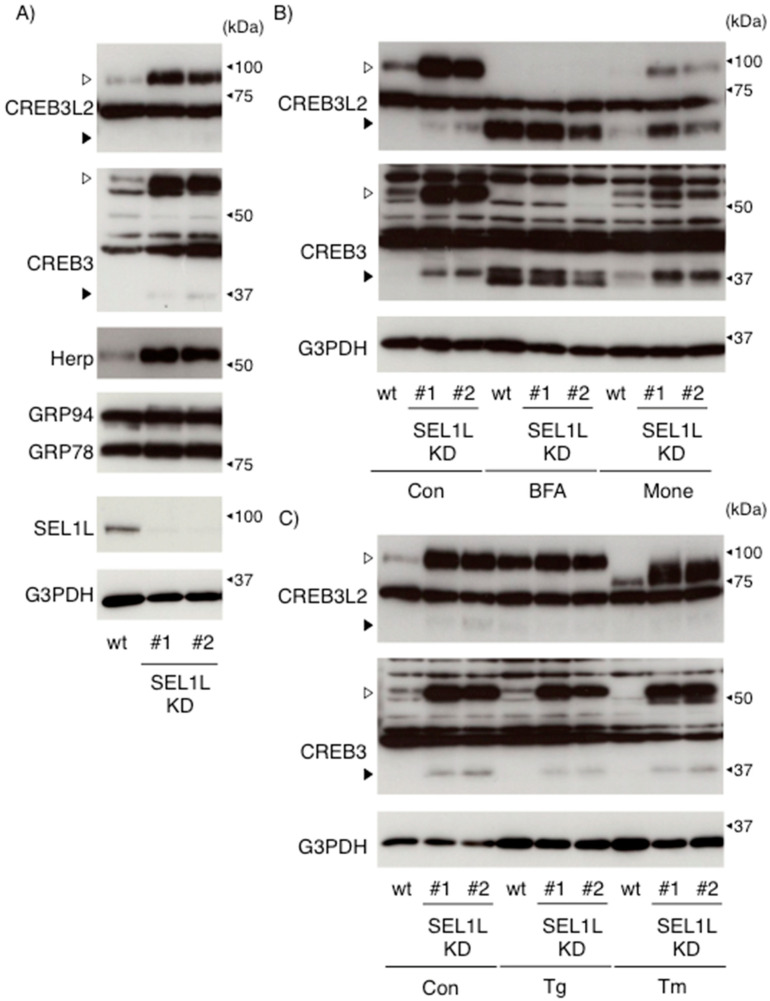

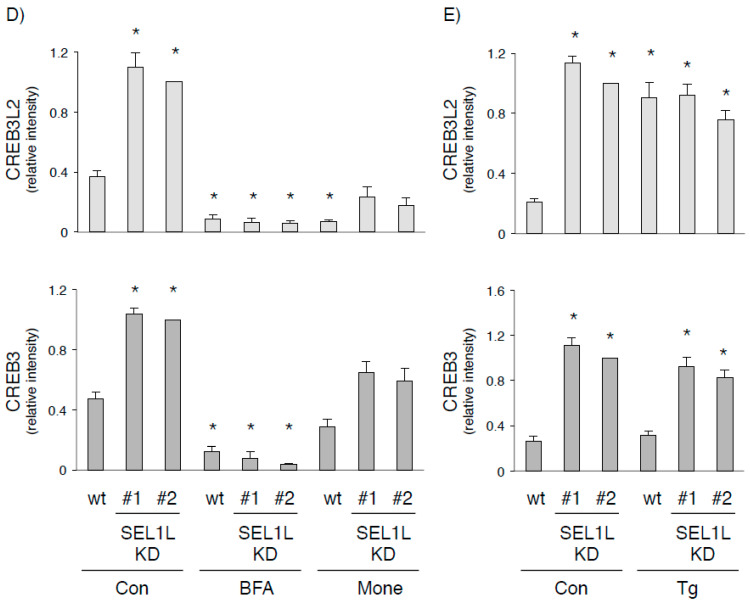
SEL1 deficiency increased CREB3 and CREB3L2 protein expression in HEK293 cells. (**A**) Wild-type (wt) and SEL1L-deficient (SEL1L-KD) HEK293 cells were cultured (**A**) and treated with BFA (0.5 μg/mL), monensin (1 μM), Tg (0.01 μM), Tm (2 μg/mL), or vehicle for 6 h (**B**–**E**). The expression of the indicated protein was detected as described in the Materials and Methods. Open and filled arrowheads indicate full-length mature and cleaved CREB3 and CREB3L2 proteins, respectively. Representative results of three independent cultures are shown. (**D**,**E**) The relative amounts of full-length CREB3 and CREB3L2 were evaluated as described in the Materials and Methods. The level of each full-length form in untreated SEL1L-deficient cells (#2) was considered “1”. Each value represents the mean ± SEM from three independent cultures. Values marked with asterisks are significantly different from value of untreated wild-type cells (* *p* < 0.05).

Treatment with cycloheximide (CHX) and MG132 revealed the rapid degradation of both endogenous CREB3 and CREB3L2 proteins by the proteasome pathway (Figure 1C). Interestingly, the molecular sizes of full-length CREB3 and CREB3L2 were slightly reduced following MG132 treatment compared with untreated control HEK293 cells. Protein homeostasis within the ER is strictly surveyed, and accumulated abnormal and/or unfolded proteins are selectively removed from the ER and degraded by the proteasome [32]. To date, various ER-associated protein degradation (ERAD) components that form a complex around the ER membrane have been identified, and their coordinated actions in regulating the ERAD machinery have been described [18,19,20,33,34]. Regarding the ATF6/CREB3 family, we and others have documented a crucial role of SEL1L in regulating ATF6, CREB3, and CREB3L3 (CREBH) protein expression [17,35,36]; however, no study has directly compared the effects of ERAD impairment on ATF6/CREB3 family protein expression. We then compared the expression of the CREB3 and CREB3L2 proteins in wild-type and SEL1L-deficient cells under several conditions. As shown in Figure 2A, two SEL1L-deficient cell lines established using different gRNAs expressed a large amount of full-length CREB3 and CREB3L2 proteins under resting conditions, as expected. In accord with our previous report, expression of Herp protein was also elevated in two SEL1L-deficient cells [17,34]. Interestingly, we observed a slight increase in the level of each cleaved form in both SEL1L-deficient cell lines without any stimuli when we exposed the detection film for a longer time (Figure 2). Since the stability of each cleaved form predominantly localizing in the nucleus is unlikely to be directly regulated by the ERAD machinery, including SEL1L, accumulated full-length CREB3 and CREB3L2 might be subject to spontaneous cleavage. Another possibility is that certain signaling pathways induced by this SEL1L deficiency might trigger their cleavage.

Next, we evaluated the expression and processing of CREB3 and CREB3L2 following treatment with BFA, monensin, Tg, or Tm. BFA treatment significantly depleted both full-length forms in wt and SEL1L-deficient cells in parallel to an increase in the levels of the cleaved forms (Figure 2B,D). In contrast, the monensin treatment depleted almost all of the full-length CREB3L2 protein only in wt HEK293 cells. Interestingly, the difference in levels of the cleaved form observed after the monensin treatment between wt and SEL1L-deficient cells was approximately proportional to the change in the level of each full-length form, although the BFA-induced cleavage of CREB3 and CREB3L2 was comparable. Regarding the degradation of the nuclear localizing N-terminal halves of ATF6/CREB3 family proteins, Fbxw7, a ubiquitin ligase, is reported to facilitate CREB3L1 and CREB3L2 degradation through the recognition of their conserved regions [37]; however, other ATF6/CREB3 family proteins lack this unique region. Under the current conditions, both the cleaved CREB3 and CREB3L2 proteins behaved similarly after the BFA/monensin treatment and SEL1L deficiency. Therefore, common regulatory mechanisms for the expression of each cleaved form might exist.

Tg treatment significantly upregulated full-length CREB3L2 in wt HEK293 cells and abolished the difference due to SEL1L deficiency (Figure 2C,E). Since the CREB3L2 promoter has only been characterized [38], further studies are needed to determine whether Tg or SEL1L deficiency increase the level of the CREB3L2 protein through a transcriptional and/or translational mechanism. Kondo et al. reported that among the three canonical ER stress sensors, the PERK pathway does not contribute to Tg-induced CREB3L2 expression; however, the contributions of other ER stress sensors, ATF6 and IRE1, remain to be determined [3,6,28,29]. This result also implies that a certain upper limit to CREB3L2 protein expression in HEK293 cells might exist. On the other hand, Tm treatment depleted only full-length CREB3 in wt cells and shifted the molecular weights of CREB3 in SEL1L-deficient cells and CREB3L2 in both cell lines, although the degree of the mobility shift in these proteins differed. These results indicate the importance of N-glycosylation of these proteins; their stability inside the ER might differ, although the mechanism by which N-glycosylation contributes to this protein stability is unclear.

**Figure 3:**
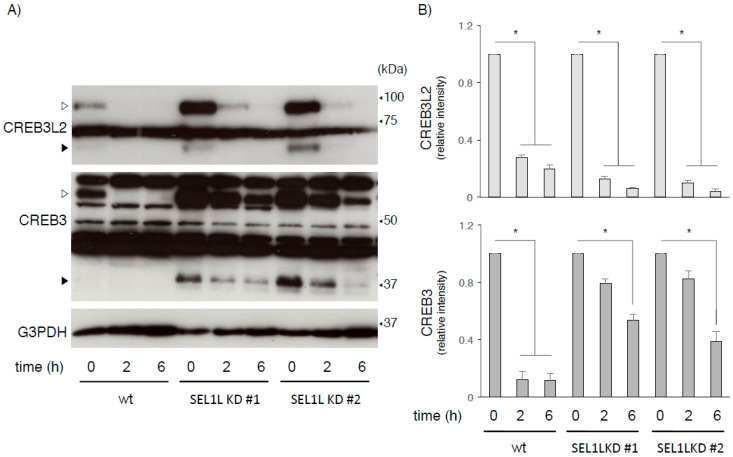
Comparison of the stability of the CREB3 and CREB3L2 proteins in HEK293 cells. (**A**) Wild-type (wt) and SEL1L-deficient HEK293 cells were treated with CHX (20 μg/mL) for the indicated times. The expression of the indicated proteins in wt and SEL1L-deficient cells was detected. Open and filled arrowheads indicate full-length mature and cleaved CREB3 and CREB3L2 proteins, respectively. Representative results of three independent cultures are shown. (**B**) The relative amounts of full-length CREB3 and CREB3L2 in wt and SEL1L-deficient cells were evaluated as described in the Materials and Methods. The amount of each protein in wt and SEL1L-deficient cells without CHX treatment was considered “1”. Each value represents the mean ± SEM from three independent cultures. Values marked with asterisks are significantly different between the indicated groups (* *p* < 0.05).

We further compared the stability of CREB3 and CREB3L2 between wt and SEL1L-deficient cells following CHX treatment. As shown in Figure 3, the CHX treatment significantly decreased CREB3 and CREB3L2 levels in both wt and SEL1L-deficient cells, but the disappearance of full-length CREB3L2 occurred more rapidly than full-length CREB3. Since both full-length forms accumulated in response to MG132 treatment and SEL1L deficiency, these differences were unexpected. Kondo et al. previously reported that the stability of the full-length ATF6 and CREB3L2 proteins in HeLa cells was quite different [39]. Therefore, degradation of the ATF6/CREB3 family proteins within the ER might not be uniformly regulated by SEL1L-dependent/independent processes. Other unstable proteasome substrates might also stabilize full-length CREB3L2 but not CREB3 within the ER in these HEK293 cells. An intriguing approach would be to compare the stability of each ATF6/CREB3 family protein simultaneously, although some CREB3 family proteins are expressed in restricted cells and tissues [1,2,3,4,5,6,7,8].

**Figure 4:**
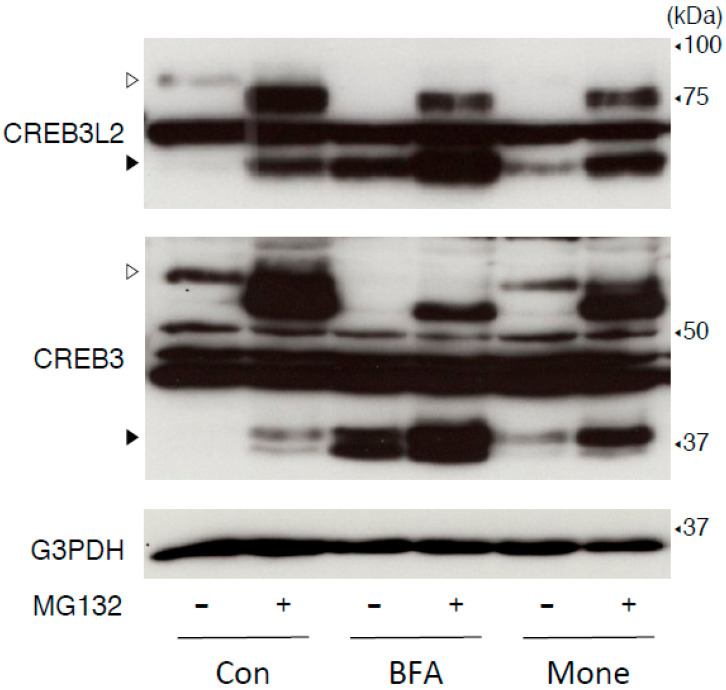
Effects of the MG132 treatment on the BFA- and monensin-induced processing of CREB3 and CREB3L2 proteins in HEK293 cells. HEK293 cells were treated with BFA (0.5 µg/mL), monensin (1 µM), or vehicle, in the presence or absence of MG132 (10 µM), for 8 h. The expression of the indicated protein was detected as described in the Materials and Methods. Open and filled arrowheads indicate full-length mature and cleaved CREB3 and CREB3L2 proteins, respectively. Representative results of three independent cultures are shown.

**Figure 5:**
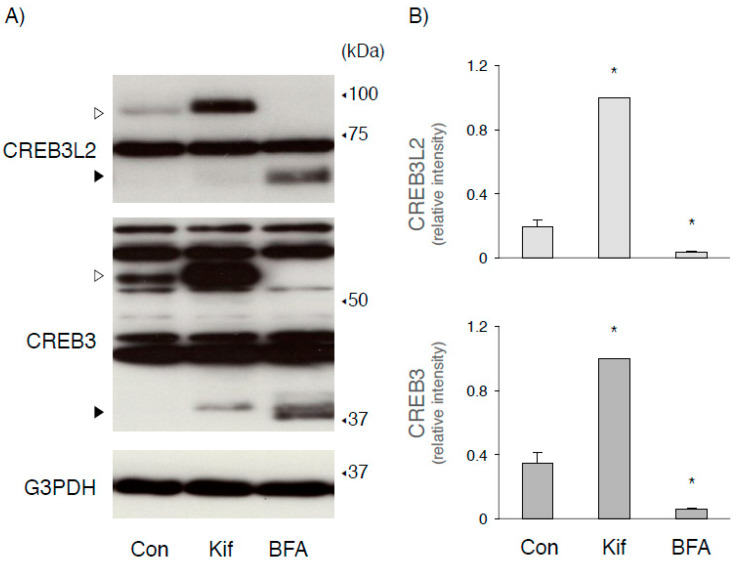
Treatment with kifunensine increased the levels of the CREB3 and CREB3L2 proteins in HEK293 cells. (**A**) HEK293 cells were treated with kifunensine (Kif, 5 µg/mL), BFA (0.5 µg/mL), or vehicle for 8 h. The expression of the indicated protein was detected as described in the Materials and Methods. Open and filled arrowheads indicate full-length mature and cleaved CREB3 and CREB3L2 proteins, respectively. Representative results of four independent cultures are shown. (**B**) The relative amounts of full-length CREB3 and CREB3L2 were evaluated as described in the Materials and Methods. The amount of each full-length form in cells treated with kifunensine was considered “1”. Each value represents the mean ± SEM from four independent cultures. Values marked with asterisks are significantly different from value of untreated cells (* *p* < 0.05).

Since treatment with BFA, but not monensin, depleted both full-length CREB3 and CREB3L2, we treated wild-type cells with each reagent in the presence or absence of MG132 (Figure 4). Cotreatment with MG132 increased the expression of CREB3 and CREB3L2; however, the molecular weights of each full-length form were slightly lower than those detected under resting control conditions. These increases induced by MG132 treatment may be attributed to two factors: one is that nascent unglycosylated full-length CREB3 and CREB3L2 proteins are quickly degraded without maturation. The other possibility is that they might correspond to deglycosylated forms removed from the ER, followed by their proteasomal degradation. We are currently unable to discriminate between the two, but these results suggest that a large amount of CREB3 and CREB3L2 proteins are continuously translated and then degraded. On the other hand, treatment with kifunensine, an α-mannosidase inhibitor [40], significantly increased levels of the full-length mature CREB3 and CREB3L2 proteins in wild-type HEK293 cells, suggesting that the effect of kifunensine on CREB3 and CREB3L2 protein expression was different from MG132 (Figure 5). Since mannose trimming is required to deliver glycosylated unfolded proteins to the ERAD machinery [32,40], this finding appears to be consistent with the results observed in SEL1L-deficient cells (Figure 2).

**Figure 6:**
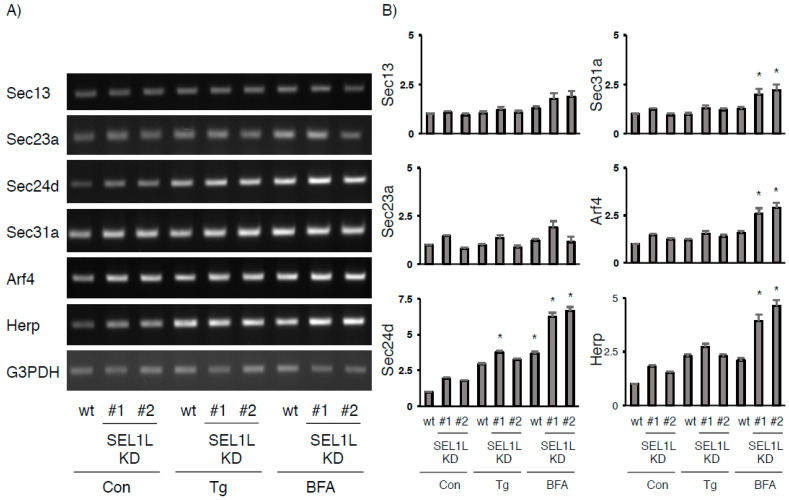
Effects of SEL1L deficiency on CREB3- and CREB3L2-regulated gene expression in HEK293 cells. (**A**) Wild-type (wt) and SEL1L-deficient HEK293 cells were treated with Tg (0.01 µM), BFA (0.5 µg/mL), or vehicle for 6 h. The expression of the indicated mRNAs in wt and SEL1L-deficient cells was detected as described in the Materials and Methods. Representative results of four independent cultures are shown. (**B**) The relative amount of each mRNA in wt and SEL1L-deficient cells was evaluated as described in the Materials and Methods. The amount of each mRNA in wt HEK293 cells without treatment was considered “1”. Each value represents the mean ± SEM from four independent cultures. Values marked with asterisks are significantly different from values value of untreated wild-type cells (* *p* < 0.01).

To date, some experimental organisms deficient in CREB3L2 have revealed a certain role for CREB3L2 in controlling the secretory capacity [13,15,16]. Ishikawa et al. documented that a subset of Sec family genes is regulated by CREB3L2 during specific developmental stages in medaka fish [41]. On the other hand, only a few genes regulated by CREB3 (e.g., Herp and Arf4) have been reported [26,42]. As shown in Figure 1 and Figure 2, SEL1L deficiency spontaneously induced the production of a small amount of each cleaved form. On the other hand, excessive cleavage of these proteins was triggered by BFA, but not Tg. Then, we tried to assess the expression of these genes in wt and SEL1L-deficient cells under three distinct conditions (Figure 6). The levels of the Sec24d, Arf4, and Herp mRNAs were slightly increased by SEL1L deficiency though their increase was not significant compared with untreated wild-type HEK293 cells. In contrast, the effects of BFA treatment on the Sec24d, Sec31a, Arf4, and Herp mRNAs in the SEL1L-deficient cells were significant. In contrast, the Sec23a mRNA was not upregulated under each condition, although both Sec23a and Sec24d possess the CRE-like region specifically recognized by CREB3L2 [14,43]. On the other hand, the Herp and Arf4 genes are reported to possess the CREB3 recognition element in their promoters [26,42,44]. In particular, CREB3 is documented to activate Herp gene transcription through ERSE-II in the Herp promoter [42,44], which is recognized by ATF6 and sXBP1 [45]. However, our preliminary experiments showed that Herp expression in CREB3-deficient cells treated with or without BFA was comparable to parental wild-type cells (unpublished data). Since SEL1L deficiency also stabilizes ATF6 [17,35], we speculated that ERAD abnormalities due to SEL1L deficiency trigger multiple signaling pathways concomitant with aberrant protein accumulation within the ER. Therefore, it is intriguing to elucidate the ER/Golgi stress responses and cell viability in the SEL1L deficient cells. In addition, these studies might give clues to how CREB3 and CREB3L2 contribute to the expression of each Sec family member, Arf4, and Herp.

Taken together, both CREB3 and CREB3L2 are SEL1L-regulated ERAD substrates in HEK293 cells; however, the signals triggering their cleavage are distinct from canonical ER stress-inducing stimuli, and their regulation is slightly different. Therefore, studies aiming to determine the specific structures of CREB3 and CREB3L2 within their transmembrane and ER luminal regions that respond to these unique stimuli would be intriguing. In addition, the identification and characterization of precise target genes of CREB3 and CREB3L2 will help clarify the whole picture of the Golgi stress response.

## 3. Materials and Methods

### 3.1. Materials

Thapsigargin (Tg), tunicamycin (Tm), brefeldin A (BFA), cycloheximide (CHX), and kifunensine (Kif) were obtained from Sigma-Aldrich (St. Louis, MO, USA). Monensin (Mone) and nigericin (Nigr) were purchased from Abcam (Cambridge, UK). MG132 (MG) and concanamycin A (CMA) were obtained from Peptide Institute (Osaka, Japan) and Wako (Osaka, Japan), respectively.

### 3.2. Establishment of SEL1L-Deficient HEK293 Cells

SEL1L-deficient HEK293 cells were established using the CRISPR/Cas9 system as described previously [17,46,47]. Briefly, a donor gene encoding the human SEL1L N-terminus (70 bp) in a pGL3-derived vector, together with constructs for each crRNA and hCas9, was transfected into HEK293 cells, and cells were selected with the appropriate concentrations of puromycin as described previously [17,46]. The target sequences of each gRNA were as follows: 5′-GAGCTTGGCCTCGGCGTCCT-3′ (SEL1L KD#1), 5′-GCAGCAGCGTCAGCCCTATC-3′ (SEL1L KD#2). 

### 3.3. Cell Culture and Treatment

HEK293 cells were maintained in Dulbecco’s modified Eagle’s minimum essential medium containing 5% fetal bovine serum. Cells were seeded into 12-well plates and treated with Tg (0.01 µM), Tm (2 µg/mL), BFA (0.5 µg/mL), Mone (1 µM), Nigr (0.2 µM), CMA (50 nM), MG132 (10 µM), CHX (20 µg/mL), Kif (5 µg/mL), or vehicle for the indicated times to detect the expression of the indicated genes and proteins using RT-PCR and immunoblotting, respectively.

### 3.4. Western Blot Analysis

The amounts of the indicated proteins in the cell lysates were detected as previously described [10,17,46]. The cells were lysed with homogenization buffer (20 mM Tris-HCl (pH 8.0) containing 137 mM NaCl, 2 mM EDTA, 10% glycerol, 1% Triton X-100, 1 mM PMSF, 10 µg/mL leupeptin, and 10 µg/mL pepstatin A). After the protein concentration was determined, an equal volume of 2× sodium dodecyl sulfate (SDS)-Laemmli sample buffer (62.5 mM Tris-HCl (pH 6.8), 2% SDS, 10% glycerol, and 12% 2-ME) was added to each cell lysate. Equal amounts of cell lysates were separated on 8-15% SDS-polyacrylamide gels and immunoblotted onto polyvinylidene difluoride membranes (Millipore, Burlington, MA, USA). The primary antibodies against CREB3 (Proteintech, Rosemont, IL, USA, antibody dilution 1:1500), CREB3L2 (Proteintech, antibody dilution 1:1000), GADD153 (Santa Cruz Biotech, Dallas, Texas, USA, antibody dilution 1:1000), SEL1L (Abcam, antibody dilution 1:1500), LC-3 (MBL, Nagoya, Japan, antibody dilution 1:3000), and G3PDH (Acris, San Diego, CA, USA, antibody dilution 1:5000) were used, and each membrane was incubated at 4 °C overnight. The horseradish peroxidase (HRP)-labeled antibodies against mouse and rabbit IgG (Cell Signaling Technology, Beverly, MA, USA, antibody dilution 1:2000) were used as the secondary antibodies, and each membrane was incubated at room temperature for 90 min. The membranes were incubated with enhanced chemiluminescence reagent (GE Healthcare, Buckingghamshire, UK) and exposed to high-performance chemiluminescence film (GE Healthcare, Buckingghamshire, UK) for appropriate times to detect antigen-antibody complexes. The experiments were repeated to confirm reproducibility. The expression level of each protein was analyzed using the ImageJ software (National Institutes of Health), and the relative amount of each protein was normalized as described in each figure legend [46].

### 3.5. Reverse Transcription Polymerase Chain Reaction

The expression level of each gene was estimated using RT-PCR. First, total RNA was extracted from cells lysed with TRI reagent (Molecular Research Center, Cincinnati, OH, USA), and equal amounts of total RNA from each sample were converted to cDNAs by reverse transcription using random nine-mers to prime SuperScript III Reverse Transcriptase (RT) (Life Technologies, Waltham, MA, USA) as previously described [17,46]. Each cDNA sample was added to a PCR mixture for amplification (Taq PCR kit, Takara, Shiga, Japan). The following PCR primers were used in this study: Arf4 sense primer, 5′-GTCACCACCATTCCTACCAT-3′, Arf4 antisense primer, 5′-TACGAAGAGACTGAAGCCCT-3′; Herp sense primer, 5′-AGGGAAGTTCTTCGGAACCT-3′, Herp antisense primer, 5′-TCAGCTGGAAACTGGTTGTG-3′; Sec13 sense primer, 5′-AAGCCTCATAGACCACCCATCG-3′, Sec13 antisense primer, 5′-CAGGTCCAAATGAACACACG-3′; Sec23a sense primer, 5′-AGCATTCAAGTGGGCAGAGA-3′, Sec23a antisense primer, 5′-TCCAGCCACCTAAGCACATCTG-3′; Sec24d sense primer, 5′-CCTCTTTCCTAGTCAGTATGTG-3′, Sec24d antisense primer, 5′-AACTCCACGGTCACTGCCTT-3′; Sec31a sense primer, 5′-CATGGGAGGTAGCACAGATG-3′, Sec31a antisense primer, 5′-AGTTGGTCTGATCGGCTGAGG-3′; G3PDH sense primer, 5′-ACCACAGTCCATGCCATCAC-3′, and G3PDH antisense primer, 5′-TCCACCACCCTGTTGCTGTA-3′. The typical reaction cycling conditions were 30 sec at 96 °C, 30 sec at 58 °C, and 30 sec at 72 °C. The results represent 21–28 cycles of amplification; then, the products were separated by electrophoresis on 2.0% agarose gels and visualized using ethidium bromide. The expression level of each mRNA was analyzed using the ImageJ software (National Institutes of Health), and the relative amount of each mRNA was calculated based on the G3PDH value obtained from the identical cDNA. The mRNA expression of each cDNA was normalized to the values obtained from the untreated wt HEK293 cells [46].

### 3.6. Statistical Analysis

The results are expressed as the mean ± SEM. Statistical analyses were carried out using one-way ANOVA followed by Tukey–Kramer test.

## Data Availability

Data sharing is not available to this article.
